# Supplementary material for: Interaction dynamics between innate and adaptive immune cells responding to SARS-CoV-2 vaccination in non-human primates
Source: Nat Commun. 2023 Dec 2;14:7961. doi: 10.1038/s41467-023-43420-x (PMC10693617; doi:10.1038/s41467-023-43420-x)
Supplement: Supplementary file 1 — Supplementary Information [file 41467_2023_43420_MOESM1_ESM.pdf]

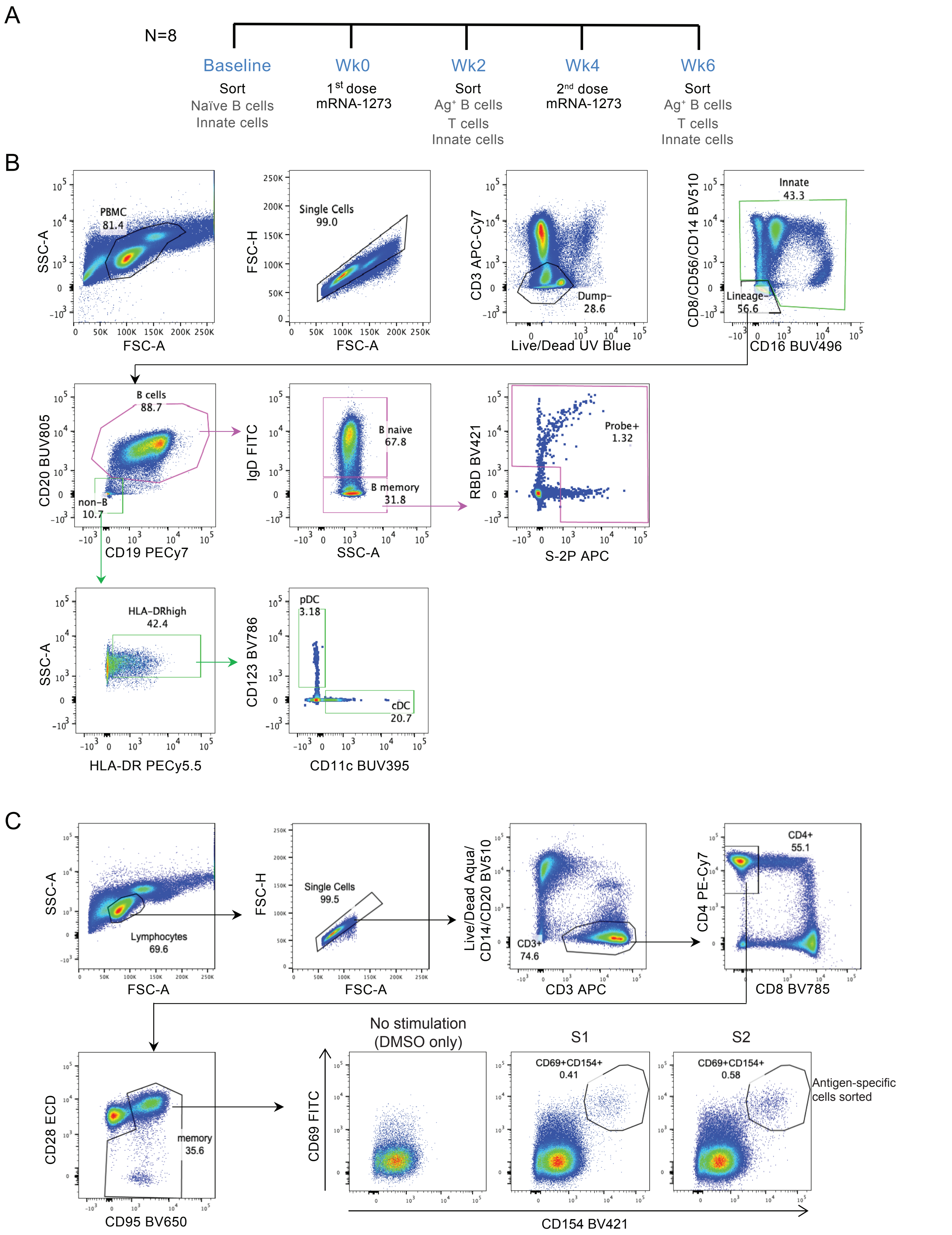

Fig. S1. Study design and sort strategies. (A) Vaccination schedule and timeline of sample collection for sort of each cell type. **(B)** Sorting strategy for B cells (pink gates) explored on Fig 1 and innate cells (green gates) explored on Fig 6. **(C)** Sorting strategy for non-specific T cells (memory CD4 T cells from DMSO control) and antigen-specific T cells explored on Fig 4.

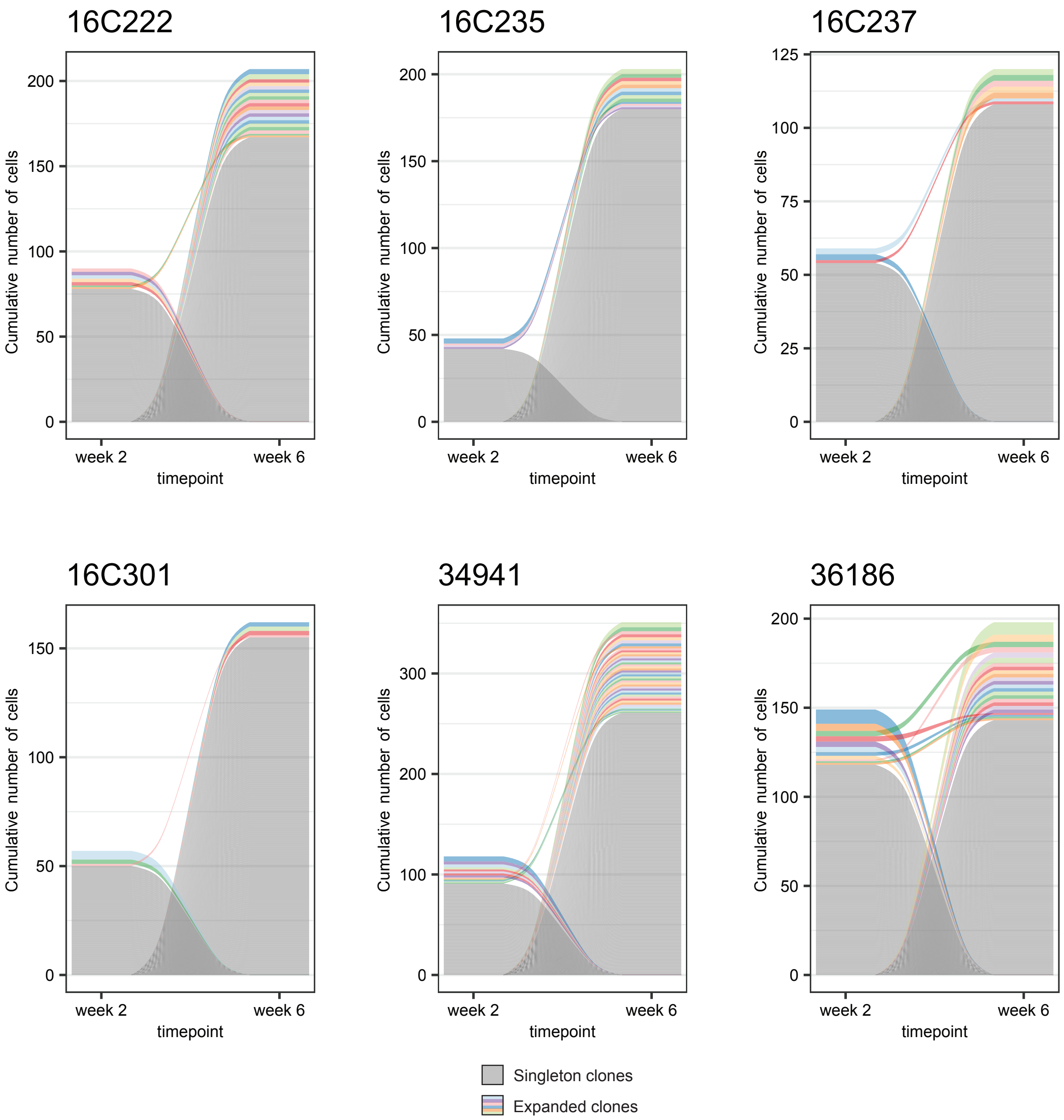

Fig. S2. B cell clonal dynamics of 6 animals. Alluvial plots, with grey lines representing singleton B cells and colored lines represent expanded lineages. Lines that start or end at zero, including all singleton, indicate B cell lineages that were observed in only a single time point. Colored lines that span the graph represent lineages found at both time points. The thickness of each line at either side of the panel is proportional to the number of cells in that lineage at the corresponding time point, with the cumulative number of cells in all lineages indicated on the y-axis. A total of 521 cells from week 2 and 1,241 cells from week 6 were analyzed. No plot is shown for animal 16C303, for which no week 6 IG sequences are available.

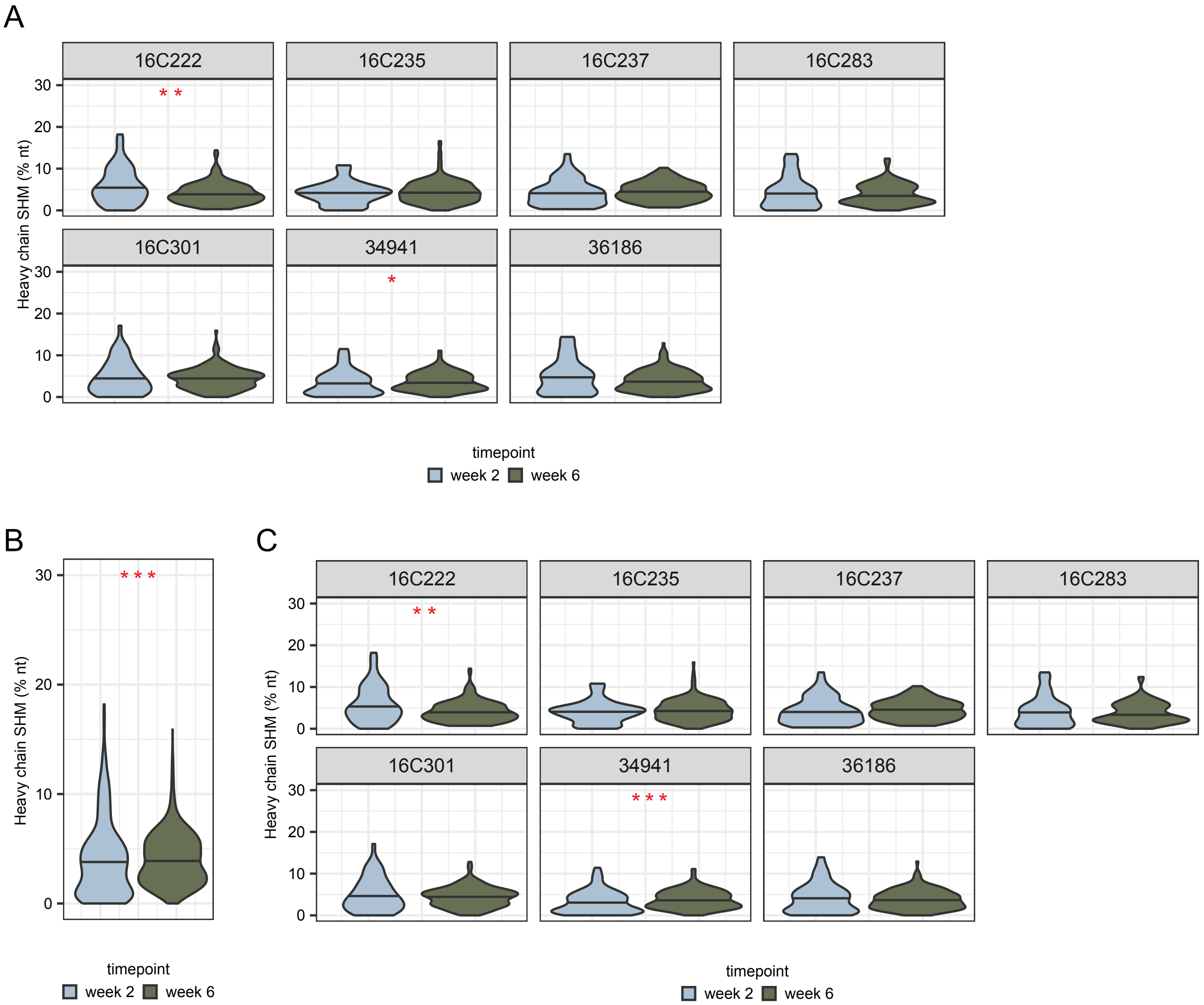

Fig. S3. Additional analysis of heavy chain somatic hypermutation (SHM). (A) Same data as shown in Fig. 2B, divided by animal. N=604 cells at week 2 and 1,393 cells at week 6. 92 cells from animal 16C303 at week 2 are not included, as no matching week 6 data were available from that animal. Distributions were compared using a 2-sided unpaired Wilcoxon test. *P* = 0.0025 for 16C222 and 0.019 for 34941. **(B)** Heavy chain SHM down-sampled to one cell per lineage to account for possible confounding effects due to correlations in SHM among cells in the same lineage. N=641 lineages at week 2 and 1,238 lineages at week 6. Distributions were compared using a 2-sided unpaired Wilcoxon test, *P* = 3.8E-4. **(C)** The same data as in (B), divided by animal, except that 87 lineages from animal 16C303 at week 2 are not included. N=554 lineages at week 2 and 1,238 lineages at week 6. Distributions were com-pared using a 2-sided unpaired Wilcoxon test. *P* = 1.1E-5 for 16C235 and 7.5E-4 for 34941.

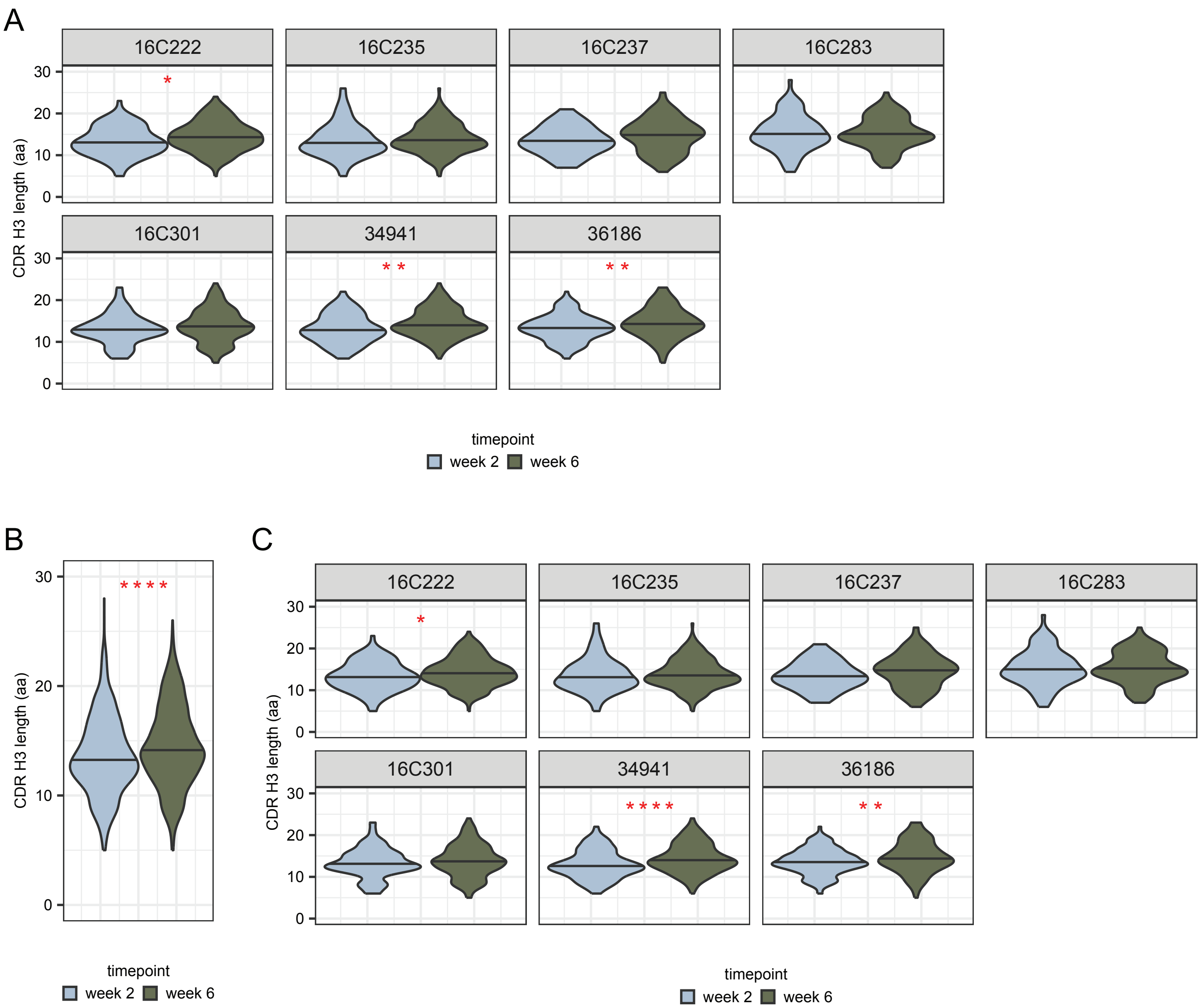

Fig. S4. Additional analysis of CDR H3 length. (A) Same data as shown in Fig. 2C, divided by animal. N=554 lineages at week 2 and 1,238 lineages at week 6. 87 lineages from animal 16C303 at week 2 are not included, as no matching week 6 data were available from that animal. Distributions were compared using a 2-sided unpaired Wilcoxon test. *P* = 0.01, 0.012, and 0.0048 for 16C222, 34941, and 36186, respectively. **(B)** CDR H3 length distribu-tions calculated using all cells in the data set. Although CDR H3 length is effectively constant within a lineage and thus not statistically independent, this shows the full post-antigen selection repertoire, including any clonal expan-sion. N=696 cells at week 2 and 1,393 cells at week 6. Distributions were compared using a 2-sided unpaired Wilcoxon test, *P* = 1.2E-7. **(C)** The same data as in (B), divided by animal, except that 92 cells from animal 16C303 at week 2 are not included. N=604 cells at week 2 and 1,393 cells at week 6. Distributions were compared using a 2-sided unpaired Wilcoxon test. *P* = 0.029, 5.6E-5, and 0.0059 for 16C222, 34941, and 36186, respectively.

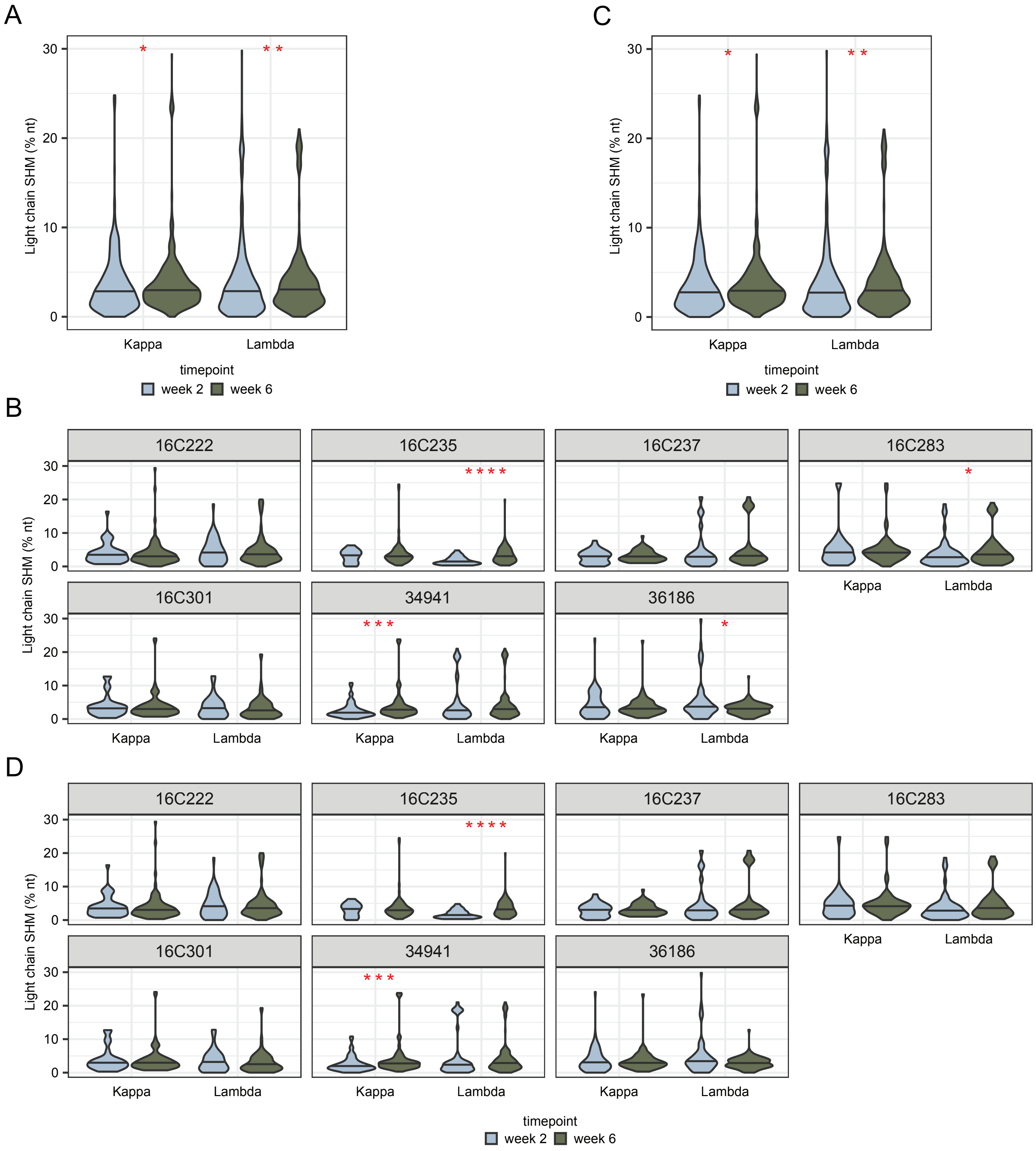

Fig. S5. Mutational burden of IG light chains. (A) Kappa and lambda light chain somatic hypermutation (SHM) of antigen-specific memory B cells. N=330 and 366 IgK and IgL sequences, respectively, at week 2 and 588 and 805 IgK and IgL sequences, respectively, at week 6. Distributions were compared using a 2-sided unpaired Wilcoxon test, $P = 0.0402$ and 0.0087 for kappa and lambda, respectively. **(B)** The same data as in (A), divided by animal. 92 cells from animal 16C303 at week 2 are not included, as no matching week 6 data were available from that animal. P = 0.028, 2.5E-4, and 0.044 for 16C283-lambda, 34941-kappa, and 36186-lambda, respectively. **(C)** Kappa and lambda chain SHM down-sampled to one cell per lineage to account for possible confounding effects due to correlations in SHM among cells in the same lineage. N=311 IgK and 330 IgL lineages at week 2 and 531 IgK and 707 IgL lineages at week 6. Distributions were compared using a 2-sided unpaired Wilcoxon test, $P = 0.016$ and 0.003 for kappa and lambda, respectively. **(D)** The same data as in (C), divided by animal, except that 43 IgK and 44 IgL lineages from animal 16C303 at week 2 are not included. N=268 IgK and 286 IgL lineages at week 2 and 531 IgK and 707 IgL lineages at week 6. Distributions were compared using a 2-sided unpaired Wilcoxon test. $P =$ 6.4E-6, 0.028, 2.5E-4, and 0.044 for 16C235-lambda, 16C283-lambda, 34941-kappa, and 36186-lambda, respectively.

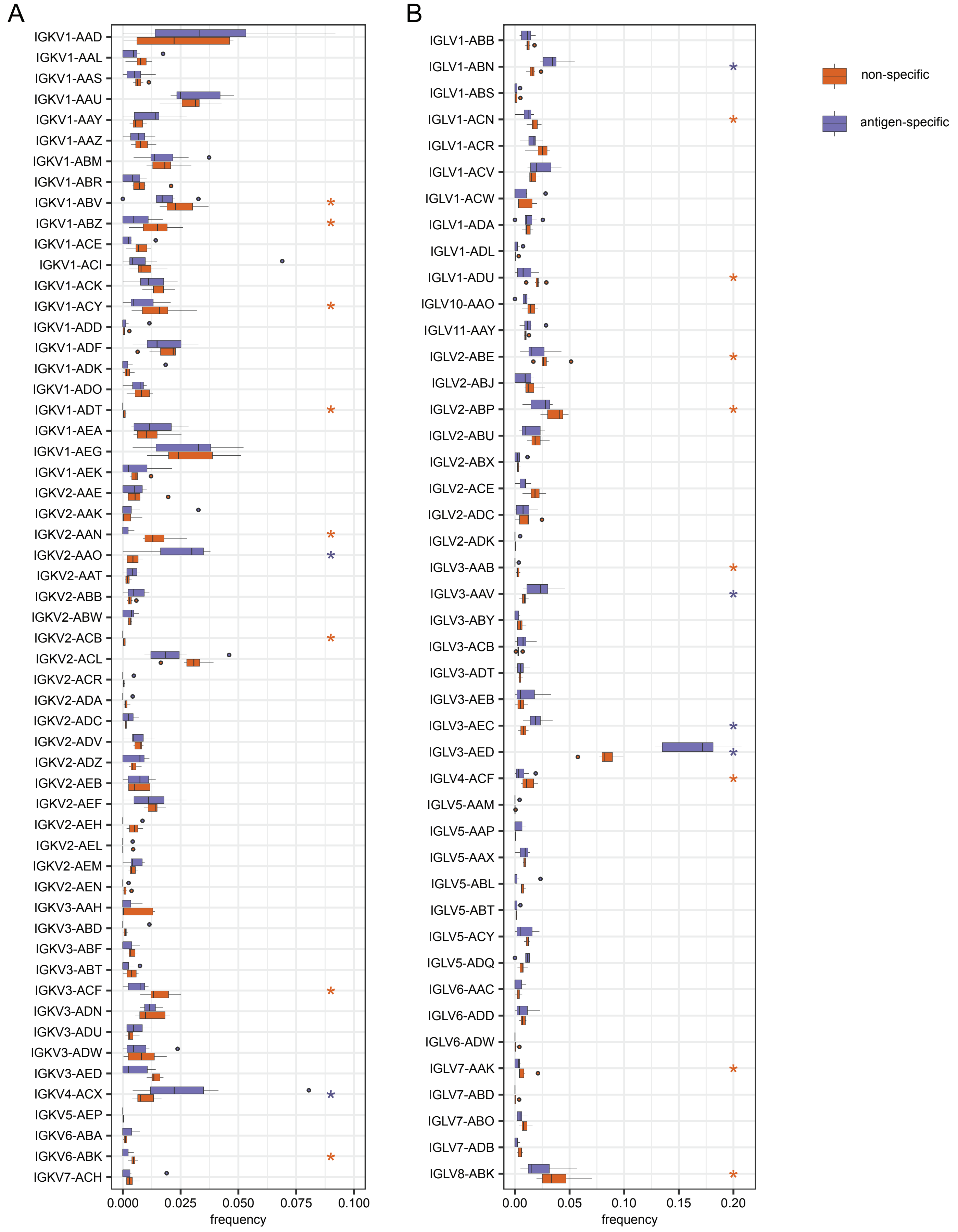

Fig S6. IG light chain V gene usage. Comparison of light chain VK **(A)** and VL **(B)** gene usage between all naïve B cells (orange) and antigen-specific memory B cells (purple) from 7 animals (animal 16C237 was excluded due to lack of naive repertoire data). N=762 IgK and 946 IgL rearrangements from antigen-specific lineages and N=13,856 IgK and 15,330 IgL rearrangements from sorted naive B cells. Boxes show the interquartile range, with the median marked as heavy horizontal band. Whisker represent the highest (lowest) datapoint within 1.5 times the interquartile range of the 75th (25th) percentile. Genes with statistically significant difference by a 2-sided paired Wilcoxon test are indicated with asterisks in the color of the up-regulated group. P = 0.016 for *IGKV2-ANN*, *IGKV3-ACF*, *IGKV6-ABK*, *IGLV1-ABN*, *IGLV1-CAN*, *IGLV2-ABP*, *IGLV3-AAB*, *IGLV3-AED*, *IGLV4-ACF*, and *IGLV7-AAK*. P = 0.031 for *IGKV4-ACX*, *IGLV2-ABE*, *IGLV3-AEC*, and *IGLV8-ABK*. P = 0.036 for *IGKV1-ADT*, *IGKV2-AAO*, and *IGKV2-ACB*. P = 0.047 for *IGKV1-ABV*, *IGKV1-ABZ*, *IGKV1-ACY*, *IGLV1-ADU*, and *IGLV3-AAV*.

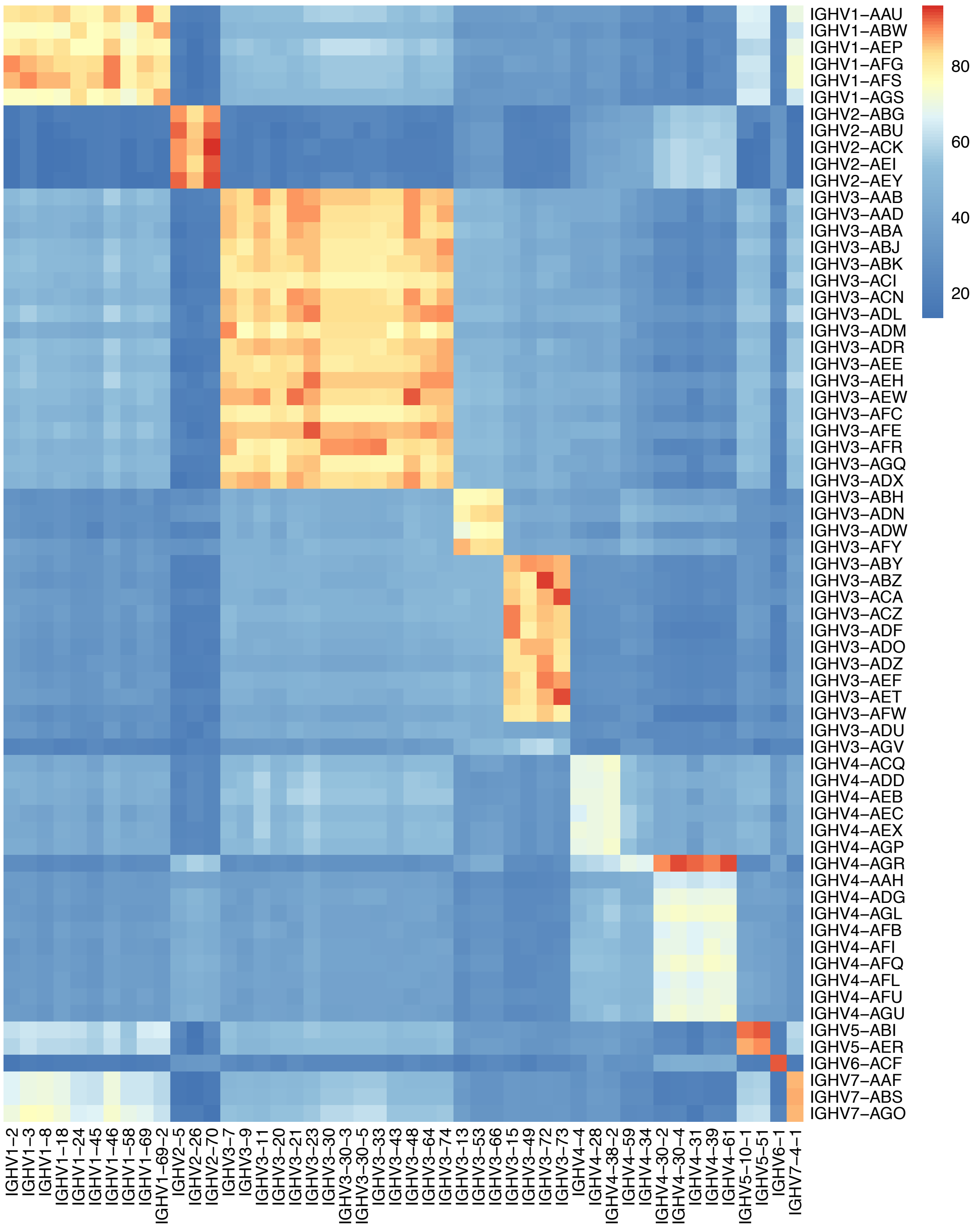

Fig. S7. Amino acid sequence homology between human and rhesus IGHV genes. Human IGHV genes are shown on the x-axis, rhesus genes are shown on the y-axis.

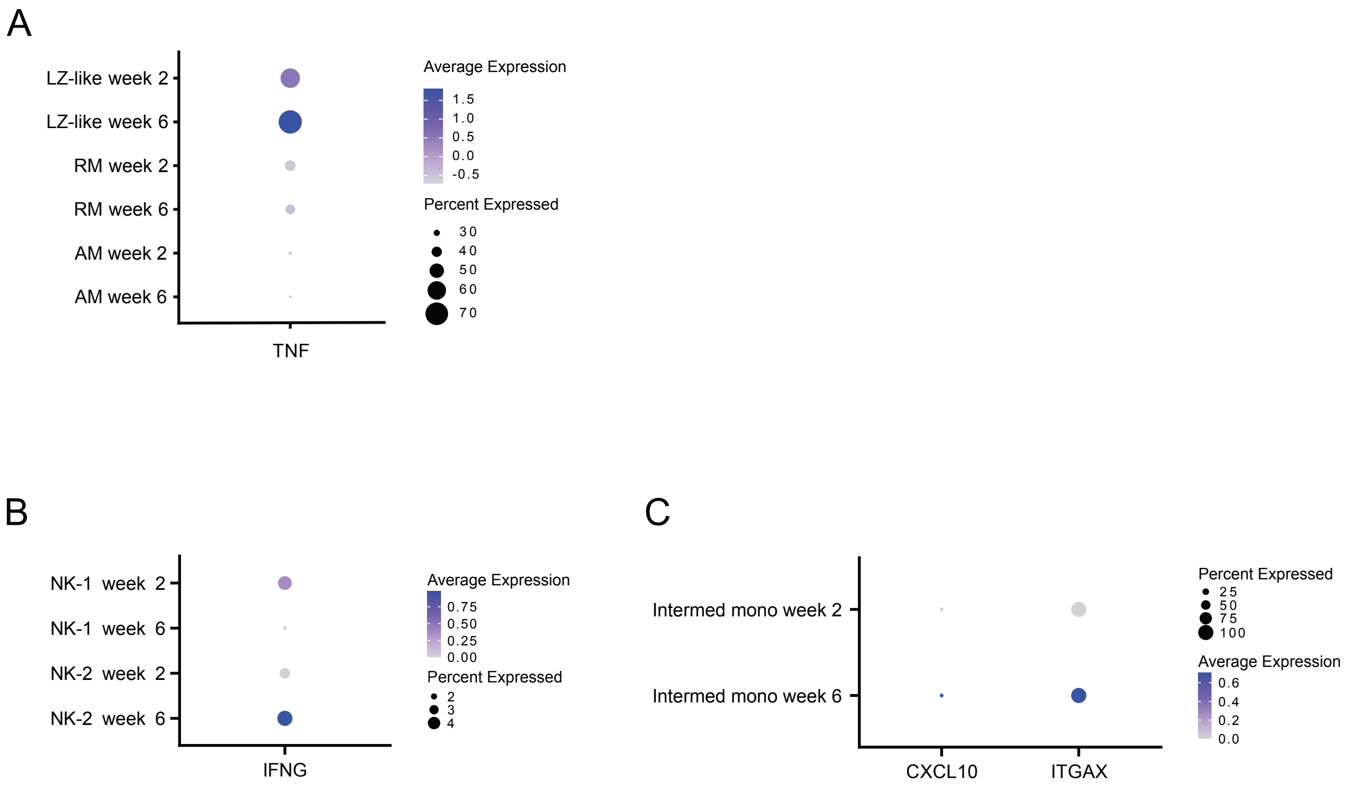

Fig. S8. Specific markers upregulated at week 6 compared to week 2. (A) LZ-like B cells upregulate *TNF* at week 6 compared to week 2. **(B)** *IFNG* is upregulated at week 6 compared to week 2 in NK-2 cells. **(C)** *ITGAX* (CD11c) is upregulated at week 6 compared to week 2 in intermediate monocytes. *CXCL10* (IP-10) expression was not detected at either time point.

Table S1. **Genes included in vaccine response signature.**

ATF3	IL1R1
BCL2A1	IL1R2
CCL2	IL6
CCL20	ITGB3
CD38	JAG1
CD69	KCNJ2
CD9	LAMP3
CLEC5A	MAFF
CXCL10	MARCO
CXCL2	NR4A2
CXCL8	NR4A3
CXCR6	OLR1
DUSP4	P2RY2
EDN1	PDGFC
EGR1	PHLDA1
EGR2	PTGS2
EGR3	REL
EREG	RGS1
F2RL1	SERPINB2
FFAR2	SLC4A4
FOSB	SOCS3
G0S2	TNFAIP6
ICAM1	TNFRSF12A
IL1A	TNFRSF21
IL1B	VEGFA

Table S2. **Antibody dilutions for innate and B cell sorting.**

Antibody	Fluorochrome	Catalog No.	Company	Clone	Dilution
IgD	FITC	2030-02	Southern Biotech	Polyclonal	1:50
CD8	BV510	301048	Biolegend	RPA-T8	1:200
CD56	BV510	318340	Biolegend	HCD56	1:200
CD14	BV510	301842	Biolegend	M5E2	1:100
CD27	BV605	302830	Biolegend	O323	1:40
CD123	BV786	564196	BD Biosciences	7G3	1:250
IgM	CF594PE	562539	BD Biosciences	G20-127	1:100
HLADR	Cy5.5PE	MHLDR18	Life Technologies	TU36	1:200
CD19	PC7	IM36284	Beckman Coulter	1A4CD27	1:20
CD11c	BUV395	744440	BD Biosciences	S-HCL-3	1:170
Live/Dead	UV blue	L34962	Invitrogen	NA	1:200
CD16	BUV496	612944	BD Biosciences	3G8	1:40
CD20	BUV805	564917	BD Biosciences	2H7	1:40
IgG	Alx700	561296	BD Biosciences	G18-145	1:40
CD3	APCCy7	557757	BD Biosciences	SP34-2	1:50

Probes

RBD	BV421
S1	BV570
S-2P	APC
NTD	BV711

Table S3. **Antibody dilutions for T cell staining.**

Antibody	Fluorochrome	Catalog No.	Company	Clone	Dilution
CD154	BV421	310824	Biolegend	24-31	1:80
CD69	FITC	310904	Biolegend	FN50	1:10
CD4	PE-Cy7	560644	BD Biosciences	L200	1:100
CD3	APC	557597	BD Biosciences	SP34-2	1:10
CD20	BV510	563067	BD Biosciences	2H7	1:20
CD95	BV650	305642	Biolegend	DX2	1:50
CD8a	BV785		Biolegend	RPA-T8	1:40
CD28	ECD	6607111	Beckman Coulter	CD28.2	1:50
CD14	BV510	301842	Biolegend	M5E2	1:20

Table S4. **Ig enrichment primers.**

**Primer**	**Sequence**
IgA_REV	GAGGCTCAGCGGGAAGACCTTGGGGCTGGTCGG
IgG_REV	GCCAGGGGGAAGACCGATGGGCCCTTGGTGGA
IgK_REV	GGGTAGAAGTTATTCAGCAGGCACAC
IgL_REV	GGGTAGAAGTCACTTATGAGACACAC
