# Supplementary material for: Interaction dynamics between innate and adaptive immune cells responding to SARS-CoV-2 vaccination in non-human primates
Source: Nat Commun. 2023 Dec 2;14:7961. doi: 10.1038/s41467-023-43420-x (PMC10693617; doi:10.1038/s41467-023-43420-x)
Supplement: Supplementary file 3 — Reporting Summary [file 41467_2023_43420_MOESM3_ESM.pdf]

nature portfolio

Corresponding author(s): Seder and Douek

Last updated by author(s): Oct 20, 2023

Reporting Summary

Nature Portfolio wishes to improve the reproducibility of the work that we publish. This form provides structure for consistency and transparency in reporting. For further information on Nature Portfolio policies, see our Editorial Policies and the Editorial Policy Checklist.

Statistics

For all statistical analyses, confirm that the following items are present in the figure legend, table legend, main text, or Methods section.

n/a	Confirmed	
☐	☒	The exact sample size (*n*) for each experimental group/condition, given as a discrete number and unit of measurement
☐	☒	A statement on whether measurements were taken from distinct samples or whether the same sample was measured repeatedly
☐	☒	The statistical test(s) used AND whether they are one- or two-sided<br>*Only common tests should be described solely by name; describe more complex techniques in the Methods section.*
☒	☐	A description of all covariates tested
☐	☒	A description of any assumptions or corrections, such as tests of normality and adjustment for multiple comparisons
☐	☒	A full description of the statistical parameters including central tendency (e.g. means) or other basic estimates (e.g. regression coefficient) AND variation (e.g. standard deviation) or associated estimates of uncertainty (e.g. confidence intervals)
☐	☒	For null hypothesis testing, the test statistic (e.g. *F*, *t*, *r*) with confidence intervals, effect sizes, degrees of freedom and *P* value noted<br>*Give P values as exact values whenever suitable.*
☒	☐	For Bayesian analysis, information on the choice of priors and Markov chain Monte Carlo settings
☒	☐	For hierarchical and complex designs, identification of the appropriate level for tests and full reporting of outcomes
☒	☐	Estimates of effect sizes (e.g. Cohen's *d*, Pearson's *r*), indicating how they were calculated

Our web collection on statistics for biologists contains articles on many of the points above.

Software and code

Policy information about availability of computer code

Data collection	BD FACSDiva Software version 9.5.1 (BD Biosciences)
Data analysis	FlowJo v10.8.1 (BD Biosciences)<br>Graphpad Prism v.8.3.1, v9.4.0, v9.4.1<br>10X Genomics cellranger v3.1.0<br>R v4.0.2, v4.0.4, v4.2.1<br>Seurat v4.0.1 (https://satijablab.org/seurat)<br>DeepImpute (https://github.com/lanagarmire/deepimpute)<br>tensorflow v2.1.0<br>fgsea (https://bioconductor.org/packages/release/bioc/html/fgsea.html)<br>Trimmomatic v0.39 (http://www.usadellab.org/cms/?page=trimmomatic)<br>STAR v2.5.1b (https://github.com/alexdobin/STAR)<br>Stringtie v1.33 (https://ccb.jhu.edu/software/stringtie/)<br>BALDR (https://github.com/scharch/BALDR)<br>filterBalder.pl (https://github.com/scharch/filterBALDR)<br>SONAR (https://github.com/scharch/SONAR) v4.2<br>ggalluvial v0.12.4 (https://corybrunson.github.io/ggalluvial/)

For manuscripts utilizing custom algorithms or software that are central to the research but not yet described in published literature, software must be made available to editors and reviewers. We strongly encourage code deposition in a community repository (e.g. GitHub). See the Nature Portfolio guidelines for submitting code & software for further information.

Data

Policy information about availability of data

All manuscripts must include a data availability statement. This statement should provide the following information, where applicable:

- Accession codes, unique identifiers, or web links for publicly available datasets
- A description of any restrictions on data availability
- For clinical datasets or third party data, please ensure that the statement adheres to our policy

Hallmark pathways for gene-set enrichment analysis were downloaded from https://www.gsea-msigdb.org/gsea/msigdb/human/genesets.jsp?collection=H. All raw and processed sequencing data generated in this study have been deposited in the Gene Expression Omnibus at https://www.ncbi.nlm.nih.gov/geo/query/acc.cgi?acc=GSE232117

Research involving human participants, their data, or biological material

Policy information about studies with human participants or human data. See also policy information about sex, gender (identity/presentation), and sexual orientation and race, ethnicity and racism.

Reporting on sex and gender	not applicable
Reporting on race, ethnicity, or other socially relevant groupings	not applicable
Population characteristics	not applicable
Recruitment	not applicable
Ethics oversight	not applicable

Note that full information on the approval of the study protocol must also be provided in the manuscript.

Field-specific reporting

Please select the one below that is the best fit for your research. If you are not sure, read the appropriate sections before making your selection.

☒ Life sciences ☐ Behavioural & social sciences ☐ Ecological, evolutionary & environmental sciences

For a reference copy of the document with all sections, see nature.com/documents/nr-reporting-summary-flat.pdf

Life sciences study design

All studies must disclose on these points even when the disclosure is negative.

Sample size	We used all 8 animals in the 100ug dose group of the original study.
Data exclusions	No data was excluded.
Replication	No replicates were conducted due to limited sample availability.
Randomization	Animals were randomly assigned to various arms of the original study; here we focus on those that received the eventual clinical dose of 100ug.
Blinding	This study was not blinded, as animals were selected based on use in a previous study and no further intervention was tested. In addition, sort probes were required to be matched to the infecting variant for the analyses described to be possible.

Reporting for specific materials, systems and methods

We require information from authors about some types of materials, experimental systems and methods used in many studies. Here, indicate whether each material, system or method listed is relevant to your study. If you are not sure if a list item applies to your research, read the appropriate section before selecting a response.

Materials & experimental systems

n/a	Involved in the study	
	☒	Antibodies
☒		Eukaryotic cell lines
☒		Palaeontology and archaeology
	☒	Animals and other organisms
☒		Clinical data
☒		Dual use research of concern
☒		Plants

Methods

n/a	Involved in the study	
☒		ChIP-seq
	☒	Flow cytometry
☒		MRI-based neuroimaging

Antibodies

Antibodies used

(B cell and innate sorts)
Antibody,Fluorochrome,Catalog No.,Company,Clone,Dilution
IgD,FITC,2030-02,Southern Biotech,Polyclonal,1:50
CD8,BV510,301048,Biolegend,RPA-T8,1:200
CD56,BV510,318340,Biolegend,HCD56,1:200
CD14,BV510,301842,Biolegend,M5E2,1:100
CD27,BV605,302830,Biolegend,O323,1:40
CD123,BV786,564196,BD Biosciences,7G3,1:250
IgM,CF594PE,562539,BD Biosciences,G20-127,1:100
HLADR,Cy5.5PE,MHLDR18,Life Technologies,TU36,1:200
CD19,PC7,IM36284,Beckman Coulter,1A4CD27,1:20
CD11c,BUV395,744440,BD Biosciences,S-HCL-3,1:170
Live/Dead,UV blue,L34962,Invitrogen,NA,1:200
CD16,BUV496,612944,BD Biosciences,3G8,1:40
CD20,BUV805,564917,BD Biosciences,2H7,1:40
IgG,Alx700,561296,BD Biosciences,G18-145,1:40
CD3,APCCy7,557757,BD Biosciences,SP34-2,1:50

(T cell stimulation)
Antibody,Fluorochrome,Catalog No.,Company,Clone,Dilution
CD154,BV421,310824,Biolegend,24-31,1:80
CD69,FITC,310904,Biolegend,FN50,1:10
CD4,PE-Cy7,560644,BD Biosciences,L200,1:100
CD3,APC,557597,BD Biosciences,SP34-2,1:10
CD20,BV510,563067,BD Biosciences,2H7,1:20
CD95,BV650,305642,Biolegend,DX2,1:50
CD8a,BV785,,Biolegend,RPA-T8,1:40
CD28,ECD,6607111,Beckman Coulter,CD28.2,1:50
CD14,BV510,301842,Biolegend,M5E2,1:20

Validation

All reagent antibodies were purchased from commercial sources and were not validated further.

Animals and other research organisms

Policy information about studies involving animals; ARRIVE guidelines recommended for reporting animal research, and Sex and Gender in Research

Laboratory animals

Macaca mulatta - six animals were age 3 years at the time of initial vaccination; 36186 was age 4 years and 34941 was age 5 years.

Wild animals

No wild animals were used in the study.

Reporting on sex

Sex was balanced during the assignment of animals to each arm of the original study. Sex was not further considered in the current analyses due to lack of statistical power.

Field-collected samples

No field-collected samples were used in the study.

Ethics oversight

Experiments in animals were performed in compliance with National Institutes of Health (NIH) regulations and with approval from the Animal Care and Use Committee of the Vaccine Research Center and from Bioqual (Rockville, MD).

Note that full information on the approval of the study protocol must also be provided in the manuscript.

Flow Cytometry

Plots

Confirm that:

- [x] The axis labels state the marker and fluorochrome used (e.g. CD4-FITC).
- [x] The axis scales are clearly visible. Include numbers along axes only for bottom left plot of group (a 'group' is an analysis of identical markers).
- [x] All plots are contour plots with outliers or pseudocolor plots.
- [x] A numerical value for number of cells or percentage (with statistics) is provided.

Methodology

Sample preparation	For B and innate cell sorts, frozen rhesus macaque PBMCs were thawed into warm R10 media (RPMI + 10% Fetal Bovine Serum + 2 mL L-Glutamine, 100 U/ml penicillin and 100 µg/ml streptomycin; all reagents from Gibco) containing DNase I (MilliporeSigma), followed by one wash with R10 and one wash with FACS buffer (PBS with 2% FBS). Cells were resuspended in 100µL of Live/Dead Fixable Blue Dead Cell Stain Kit (Invitrogen, cat# L23105) diluted 1:200 in PBS for 10 min at room temperature. Cells were washed with FACS buffer and incubated for 20 min with the staining cocktail consisting of antibodies and probes.<br>For T cell sorts, frozen rhesus macaque PBMC were thawed into warm R10 media (RPMI + 10% Fetal Bovine Serum + 2 mL L-Glutamine, 100 U/ml penicillin and 100 µg/ml streptomycin; all reagents from Gibco) containing DNase I (MilliporeSigma) and rested for 1 hour at 37oC / 5% CO2. Cells were stimulated with two peptide pools corresponding to S1 and S2 of the vaccine insert SARS-CoV-2 S protein (JPT Peptide Technologies) at 2 µg/mL of each peptide for 6 hours at 37oC / 5% CO2. A DMSO only control was included for each sample. Anti-CD154 BV421(Biolegend, clone 24-31, cat# 310824) was included during the 6-hour culture and GolgiStop (BD Biosciences) was added after 2 hours of stimulation. Following stimulation, cells were washed and stained with Aqua LIVE/DEAD dye (ThermoFisher) for 10 minutes, and subsequently stained with an antibody mix
Instrument	BD FACSymphony S6 Cell Sorter
Software	BD FACSDiva Software version 9.5.1
Cell population abundance	The purity of the cells was determined by the flow cytometry using the gating strategy provided.
Gating strategy	Gating strategies and gate boundaries are shown as flow plots in the Extended Data. Briefly, for B cell sorting and analysis, cells were gated on FSC(A)xSSC(A) -> FSC(H)xFSC(A) -> CD3-LD- -> CD8-CD56-CD14-CD16- -> CD19+CD20+ -> IgD+ (for background) OR IgD- probe+ (for antigen-specific).<br>Monocytes and NK cells were sorted as FSC(A)xSSC(A) -> FSC(H)xFSC(A) -> CD3-LD- -> CD16+ OR CD8/CD14/CD56+.<br>DCs were sorted as FSC(A)xSSC(A) -> FSC(H)xFSC(A) -> CD3-LD- -> CD8-CD56-CD14-CD16- -> CD19-CD20- -> HLA-DR high -> CD11c+ (cDC) OR CD123+ (pDC).<br>Memory CD4 T cells were gated on FSC(A)xSSC(A) -> FSC(H)xFSC(A) -> CD14/CD20LD- CD3+ -> CD4+CD8+ -> CD95 high OR CD95- CD28ECD-. After stimulation, cells were gated as CD69+CD154+ (antigen specific) or taken ungated from the DMSO negative control.

- [x] Tick this box to confirm that a figure exemplifying the gating strategy is provided in the Supplementary Information.
